# Surdos que se comunicam por meio da língua de sinais: a complexidade do acesso aos serviços de saúde

**DOI:** 10.1590/0102-311XPT00211524

**Published:** 2025-10-03

**Authors:** Rafael Rodrigues de Moraes, Laura Sabrinny de Sá Pereira, Priscila Maria Stolses Bergamo Francisco, Giovana Astolfi Pico, Nubia Garcia Vianna

**Affiliations:** 1 Universidade Estadual de Campinas, Campinas, Brasil.; 2 Faculdade de Ciências Médicas, Universidade Estadual de Campinas, Campinas, Brasil.; 3 Faculdade de Ciências Farmacêuticas, Universidade Estadual de Campinas, Campinas, Brasil.

**Keywords:** Perfil Epidemiológico, Perda Auditiva, Língua de Sinais, Acesso aos Serviços de Saúde, Epidemiological Profile, Hearing Loss, Sign Language, Access to Health Services, Perfil Epidemiológico, Pérdida Auditiva, Lengua de Signos, Acceso a los Servicios de Salud

## Abstract

O objetivo foi analisar o acesso à saúde de indivíduos surdos que se comunicam por meio da Língua Brasileira de Sinais (Libras), com 18 anos ou mais, moradores na Região Metropolitana de Campinas, São Paulo, Brasil, comparando o sistema público com o privado. Trata-se de estudo epidemiológico descritivo transversal. O instrumento de coleta de dados foi um formulário on-line acessível em Libras. Foram feitas análises estatísticas de regressão logística, quadros de contingência e de frequência e georreferenciamento. Participaram 316 indivíduos, cuja maioria considerou ruim a comunicação com profissionais da saúde, seja da rede pública (64,3%) ou da rede privada (67,6%). A probabilidade de haver uma boa comunicação é somente de 40,47%, no melhor dos casos (usuário da rede pública e com autoavaliação positiva de seu estado de saúde). Surdos usuários de Libras e unicamente da rede pública apresentaram 22% mais chances de ter uma boa comunicação com o profissional de saúde quando comparados com aqueles que frequentam a rede privada. Tanto os atendimentos nos serviços públicos de saúde quanto nos serviços privados carecem de acessibilidade linguística e comunicacional, sobretudo, pelo desconhecimento da Libras por parte dos profissionais.

## Abstract (en)

The aim of the present study was to analyze access to healthcare among deaf individuals who communicate through Brazilian Sign Language (Libras, acronym in Portuguese) 18 years of age or older residing in the Metropolitan Region of Campinas, São Paulo State, Brazil, comparing public and private healthcare systems. A descriptive cross-sectional epidemiological study was conducted. The data collection instrument was an online form accessible in sign language. Statistical analysis involved contingency and frequency tables, georeferencing, and logistic regression. Among the 316 participants, most considered communication with health professionals to be poor in both the public (64.3%) and private (67.6%) systems. The likelihood of effective communication was only 40.47% in the best-case scenario (patients in public healthcare with a positive self-assessment of their health status). Hearing impaired individuals who use Libras and only use the public healthcare system were 22% more likely to have effective communication with health professionals compared to those who use the private healthcare system. Both public and private health services lack linguistic and communication accessibility, mainly due to a lack of knowledge of Libras on the part of health professionals.

## Abstract (es)

El objetivo de este estudio fue analizar el acceso a la salud de personas sordas que se comunican por la Lengua Brasileña de Señas (Libras), mayores de 18 años, residentes en la Región Metropolitana de Campinas, São Paulo, Brasil, al comparar el sistema público con el privado. Se trata de un estudio epidemiológico, descriptivo y transversal. Para la recopilación de datos se utilizó un formulario en línea accesible en Libras. Se realizaron análisis estadísticos de regresión logística, tablas de contingencia y de frecuencia y georreferenciación. Participaron un total de 316 individuos, la mayoría de los cuales consideraron mala la comunicación con los profesionales de la salud, ya sea de la red pública (64,3%) o de la red privada (67,6%). Hubo un 40,47% de probabilidad de buena comunicación en la mayoría de los casos (usuario de la red pública y con una autoevaluación positiva de su estado de salud). Los usuarios sordos de Libras, solo de la red pública, tenían un 22% más de probabilidades de tener una buena comunicación con el profesional de la salud en comparación con los que asisten a la red privada. Tanto la atención en los servicios de salud pública como en los servicios privados carecen de accesibilidad lingüística y comunicacional, principalmente debido a la falta de conocimiento de Libras por parte de los profesionales.

## Introdução

A comunicação é uma ferramenta imprescindível na prestação de um serviço de saúde com qualidade. Falhas nesse processo afetam diretamente a relação entre profissional e paciente, favorecendo a ocorrência de erros diagnósticos, prescrições equivocadas, não adesão ao tratamento e sofrimento ^1^.

Em se tratando de indivíduos surdos usuários de Língua Brasileira de Sinais (Libras), o processo de cuidado pode ser permeado por diversas barreiras comunicacionais, tanto em relação à sua inserção no sistema de saúde quanto à sua permanência nesses serviços ^1^ ^,^ ^2^ ^,^ ^3^. Embora, pela legislação, as pessoas surdas tenham direito à comunicação acessível em estabelecimentos de saúde por profissionais capacitados para uso de Libras ou sua tradução e interpretação ^4^, essa não é a realidade da maioria dos surdos ^5^.

Os conceitos de pessoa com deficiência auditiva e pessoa surda não são equivalentes, embora se refiram às pessoas que compartilham uma diferença sensorial relacionada à audição. Enquanto a noção de deficiência é atrelada a uma limitação sensorial imposta pela perda auditiva e, portanto, relacionada aos discursos médicos, o conceito de pessoa surda ou pessoa com surdez refere-se a uma noção identitária caracterizada pelo pertencimento à comunidade surda ^6^.

Evidenciar essas diferenças é fundamental para não atribuir ao surdo a ideia de doença ou deficiência, evitando que seja visto sob rótulos socialmente impostos ^7^. Denominá-los surdos evita a imposição de estereótipos sociais, além de respeitar suas diferenças e fortalecer sua luta por direitos sociais ^7^ ^,^ ^8^. Essa perspectiva está em conformidade com o modelo socioantropológico da surdez e com o modelo social da deficiência, elaborações sociológicas que abordam a deficiência sob um ponto de vista político, como um fenômeno que não está restrito ao indivíduo, mas abrange uma organização social que marginaliza pessoas com determinados impedimentos no corpo ^9^.

Essa visão da surdez implica o compartilhamento de valores culturais, autorreconhecimento, histórias de luta social e identidade linguística relacionados à língua de sinais ^3^, com estudos nacionais e internacionais reconhecendo a comunidade surda como uma minoria cultural caracterizada pelo uso de uma língua de modalidade gestual-visual ^10^ ^,^ ^11^ ^,^ ^12^ ^,^ ^13^.

O modelo social da deficiência surge em contraponto ao modelo médico, que trata a deficiência como um fenômeno biológico, circunstanciado ao indivíduo e responsável pelas desvantagens sociais decorrentes de sua deficiência ^9^. Segundo o modelo médico, a deficiência é considerada algo indesejado a ser tratado e corrigido, porque é analisada com base nos princípios de cura, correção e assistência ^14^.

Ao abordar a questão como uma forma natural de diversidade humana e não como uma tragédia pessoal, o modelo social da deficiência deslegitima a opressão voltada a essas pessoas e, diferente do modelo médico, reivindica que a principal mudança a ser buscada para garantir sua inclusão deve ser feita na sociedade, não no indivíduo ^9^. Seguindo essa linha de raciocínio, a principal intervenção, no caso da surdez, não deve ter como alvo o indivíduo surdo, mas a sociedade.

Uma das possíveis formas de intervir é por meio de políticas públicas que levem em conta essas diferenças e, para tanto, os inquéritos epidemiológicos e demográficos são importantes ferramentas, desde que considerem as particularidades das pessoas surdas, sobretudo sua língua, para embasar o planejamento e ações que visem a uma sociedade mais acessível e inclusiva. Pessoas que partilham de uma mesma deficiência não são um grupo homogêneo. Para pessoas surdas, as narrativas e necessidades de acessibilidade em torno da surdez são diversas: que formas de comunicação compreendem, se usam Libras, se são oralizadas, se usam dispositivos auxiliares de audição, entre outros. Além da perda de audição, outras variáveis que transversalizam esses indivíduos devem ser consideradas: contato com grupos e comunidades surdas, acesso à escola bilíngue, posicionamento político, entre outras ^15^.

Todas essas questões são importantes na luta pelos seus direitos, dentre eles o direito à saúde. A garantia desse direito passa pela possibilidade de acesso aos serviços de saúde e pela qualidade de comunicação com o profissional, o que demanda conhecer as diversas formas de se subjetivar pela surdez ^16^ e as diferentes demandas de comunicação e acessibilidade ^15^.

Embora haja na literatura, nacional e internacional, estudos que abordem o acesso à saúde de pessoas surdas usuárias de língua de sinais, não foram encontradas pesquisas que contemplem uma análise comparativa do público-privado, sendo essa uma lacuna que este estudo busca preencher.

Considerando tais informações, este estudo tem como objetivo analisar o acesso à saúde de indivíduos surdos usuários de Libras, com 18 anos ou mais e moradores da Região Metropolitana de Campinas, São Paulo, Brasil, por meio da qualidade de comunicação com os profissionais em serviços públicos e privados, da sua distribuição espacial e da estrutura de serviços públicos de saúde formados pelas unidades básicas de saúde (UBS) no Município de Campinas.

## Métodos

### Delineamento e local de estudo

Trata-se de um estudo epidemiológico transversal realizado na Região Metropolitana de Campinas, entre agosto de 2021 e março de 2023. Os participantes selecionados via amostragem intencional responderam a um questionário estruturado majoritariamente em perguntas de múltipla escolha que continha os temas: autoavaliação do estado de saúde, comunicação com os profissionais de saúde das redes pública e privada, utilização de dispositivos auxiliares de audição, bem como aspectos sociodemográficos como sexo, idade, cor, escolaridade, religião, empregabilidade, estado geral de saúde, hipertensão, diabetes e hábitos de vida (tabagismo e consumo de álcool).

### Critérios de inclusão e exclusão

Para serem incluídos na pesquisa, os participantes precisavam ser uma pessoa surda, usuária de Libras, ter 18 anos ou mais e residir na Região Metropolitana de Campinas. Como critério de exclusão, foram desconsideradas as respostas de participantes não anuentes ao Termo de Consentimento Livre e Esclarecido (TCLE) e surdos não usuários de Libras.

### Coleta dos dados

A coleta foi realizada via formulário on-line da ferramenta Google Forms (https://docs.google.com/forms), escolhido pelo baixo custo, maior acessibilidade e rapidez, embora mais propenso a erros de classificação ^17^, acessível em Língua Portuguesa escrita e Libras em vídeo, testado previamente entre surdos usuários de Libras não integrantes do público-alvo da pesquisa. O formulário e os microdados anonimizados da pesquisa estão disponíveis nas línguas portuguesa, inglesa, espanhola e Libras no Repositório de Dados de Pesquisa da Universidade Estadual de Campinas (REDU/Unicamp; https://doi.org/10.25824/redu/IQX0EY).

O formulário foi amplamente divulgado nas mídias sociais, nos veículos de imprensa locais e em eventos da comunidade surda, com a ajuda da Associação de Surdos de Campinas (Assucamp) e da Central de Interpretação de Libras (CIL). A CIL é um equipamento da gestão pública municipal que disponibiliza profissionais tradutores e intérpretes de língua de sinais (TILS) à população surda como mediadores da comunicação com ouvintes, sempre que acionados, desde que a situação comunicativa ocorra em serviços municipais.

Ao todo, foram coletadas 588 respostas voluntárias, das quais 316 eram válidas segundo os critérios de inclusão e exclusão da pesquisa. A amostragem do estudo é intencional, portanto, os resultados não podem ser generalizados além da amostra.

### Análise dos dados

A análise dos dados consistiu em quatro etapas. Na primeira, a comunicação entre profissionais de saúde e os 316 participantes (Tabela 1) foi objeto de análise descritiva simples via tabelas de frequência.

**Tabela 1:** Avaliação dos participantes, usuários de Língua Brasileira de Sinais (Libras), sobre a acessibilidade linguística e comunicacional nos serviços de saúde de atendimentos gerais e específicos para demandas auditivas.

Variáveis pesquisadas	n	%
Avaliação dos participantes usuários de Libras sobre a acessibilidade linguística e comunicacional nos serviços de saúde de maneira geral		
Utiliza serviços de saúde do SUS	316	100,0
Não	61	19,3
Sim	255	80,7
Comunicação com profissionais no SUS	255	100,0
Boa, eu consigo ouvir com ajuda de aparelho ou implante e falar	8	3,1
Boa, o profissional de saúde sabe Libras	3	1,2
Boa, porque um familiar me ajuda	55	21,6
Boa, tem um intérprete que me ajuda	25	9,8
Ruim, não consigo me comunicar	65	25,5
Ruim, não sabem Libras e preciso escrever em português	50	19,6
Ruim, não sabem Libras e sou obrigado a oralizar	49	19,2
Acompanhamento do intérprete da CIL	25	100,0
Não, porque eu esqueço de pedir	1	4,2
Não, porque eu não sinto necessidade	2	8,3
Não, porque não tem CIL na minha cidade	1	4,2
Não, porque não tem intérprete livre	8	33,3
Sim	12	50,0
Não respondeu	1	-
Utiliza rede particular de saúde	315	100,0
Não	90	28,5
Sim	226	71,5
Comunicação com profissionais na rede particular	226	100,0
Boa, eu consigo ouvir com ajuda de aparelho ou implante e falar	11	4,9
Boa, o profissional de saúde sabe Libras	1	0,4
Boa, porque um familiar me ajuda	43	19,1
Boa, tem um intérprete que me ajuda	18	8,0
Ruim, não consigo me comunicar	66	29,3
Ruim, não sabem Libras e preciso escrever em português	45	20,0
Ruim, não sabem Libras e sou obrigado a oralizar	41	18,2
Não respondeu	1	-
Avaliação dos participantes sobre a acessibilidade linguística e comunicacional em serviços de saúde específicos para demandas auditivas		
Utiliza aparelho auditivo	316	100,0
Não	200	63,5
Sim	115	36,5
Não respondeu	1	-
Obteve aparelho pelo SUS	115	100,0
Não	38	33,0
Sim	77	67,0
Comunicação com profissionais no SUS com quem obteve aparelho	77	100,0
Boa, eu consigo ouvir com ajuda de aparelho ou implante e falar	8	10,4
Boa, o profissional de saúde sabe Libras	7	9,1
Boa, porque um familiar me ajuda	16	20,8
Boa, tem um intérprete que me ajuda	3	3,9
Ruim, não consigo me comunicar	15	19,5
Ruim, não sabe Libras e preciso escrever em português	10	13,0
Ruim, não sabe Libras e sou obrigado a oralizar	18	23,4
Utiliza implante coclear	316	100,0
Não	295	93,4
Sim	21	6,6
Obteve implante coclear pelo SUS	21	100,0
Não	12	57,1
Sim	9	42,9
Comunicação com profissionais no SUS com quem obteve implante coclear	9	100,0
Boa, porque um familiar me ajuda	3	33,3
Boa, tem um intérprete que me ajuda	1	11,1
Ruim, não sabe Libras e sou obrigado a oralizar	5	55,6

CIL: Central de Interpretação de Libras; SUS: Sistema Único de Saúde.

Nota: o n se altera porque, por questões éticas, nenhuma pergunta era de resposta obrigatória e, por isso, nem todas foram respondidas.

Na segunda etapa, considerando somente os 174 participantes que autodeclaram utilizar ambas as redes pública e privada de saúde e com o objetivo de atestar o quanto a comunicação entre profissionais de saúde e participantes da pesquisa concordam entre si, os dados foram analisados a partir de uma tabela de contingência (operacionalizada no artigo via mapa de calor) e medidas de concordância como o gama de Goodman-Kruskal, cuja escala vai de -1 a 1, sendo 1 um indicativo de concordância perfeita entre a qualidade de comunicação entre redes ^18^. Os dados são considerados pareados, pois cada participante avalia ambas as redes. A concordância refere-se àquelas situações nas quais o participante avaliou as redes da mesma forma, ou seja, comunicação com o profissional de saúde avaliada como boa ou ruim nas redes pública e privada.

Na terceira etapa, considerando somente os 133 participantes que autodeclararam utilizar exclusivamente uma das redes, foram usados modelos de regressão logística ^18^ para inferir quais fatores mais afetam a qualidade da comunicação, por permitirem a estimação da probabilidade de boa comunicação entre participantes e profissionais de saúde com base nas demais variáveis coletadas na pesquisa. Tanto a variável de desfecho (comunicação entre participante e profissional de saúde) quanto as covariáveis (rede, idade, sexo, escolaridade, trabalho, autoavaliação do estado de saúde, religião, cor, diabetes, hipertensão, tabagismo e consumo de álcool) foram transformadas para permitir a comparação por meio das razões de chances baseadas nos níveis de referência de cada variável do modelo.

A variável de desfecho comunicação contém níveis “Ruim” (composta por “Ruim, não consigo comunicação”; “Ruim, não sabem Libras e sou obrigado a oralizar”; “Ruim, não sabem Libras e preciso escrever português”) e “Boa” (composta por “Boa, tem intérprete que me ajuda”; “Boa, porque familiar me ajuda”; “Boa, eu consigo ouvir com ajuda de aparelho ou implante e falar” e “Boa, o profissional de saúde sabe Libras”).

As covariáveis são rede (pública e privada), idade (abaixo dos 40 anos e acima dos 40 anos), sexo (feminino e masculino), escolaridade (baixa, formada por sem escolaridade, Ensino Fundamental e Ensino Médio; e alta, formada por participantes com nível Superior completo e/ou Pós-graduação), trabalho (não e sim), cor (branca, negra e outros), religião (não e sim), autoavaliação do estado de saúde (negativa, formada por muito ruim, ruim e regular; e positiva, formada por autoavaliações boa e muito boa), diabetes, hipertensão, fumante e consumo de álcool (não e sim).

Adotou-se a parcimônia como estratégia de modelagem, privilegiando modelos de qualidade similar, porém mais simples (menos variáveis explicativas) em detrimento de modelos mais complexos. Diversos modelos, todos sem interações entre variáveis, de forma gradual e cumulativa em relação às variáveis explicativas, foram ajustados de forma aninhada, ou seja, um determinado modelo contém todas as variáveis explicativas do modelo prévio, acrescido da variável explicativa que o diferencia do anterior, permitindo, assim, a identificação, por meio do teste de razão de verossimilhanças, de quais modelos apresentavam as menores somas de resíduos do tipo deviance e, portanto, significativos ao nível de 5% no teste de razão de verossimilhanças para a comparação entre si.

Na quarta etapa de análise, de natureza descritivo-espacial e considerando somente os 142 participantes residentes em Campinas, utilizaram-se dados georreferenciados para a produção de um mapa com a proximidade e a distribuição espacial de participantes e da estrutura de serviços públicos de saúde formados pelas UBS, segundo suas áreas adscritas distribuídas em seis Distritos de Saúde (Leste, Norte, Noroeste, Sul, Sudoeste e Sudeste). O georreferenciamento dos participantes teve como base a autodeclaração do CEP residencial, a partir do qual foram obtidos os pares de latitude e longitude aproximados dos participantes da pesquisa. O georreferenciamento de UBS foi realizado com base nos pares de latitude e longitude disponíveis no Cadastro Nacional de Estabelecimentos de Saúde (CNES), versão de março/2025.

### Aspectos éticos

Adotou-se neste artigo o *checklist* CHERRIES para melhoria de qualidade em pesquisas via internet ^19^.

A pesquisa foi aprovada pelo Comitê de Ética em Pesquisa, Faculdade de Ciências Médicas, Unicamp (parecer nº 5.302.677 e CAAE 46533221.8.0000.5404) e todos os participantes tiveram acesso ao TCLE em Língua Portuguesa e Libras. Por exigência do Comitê, nenhuma das questões do formulário era vinculante, ou seja, de resposta obrigatória, por isso o número de respostas válidas varia nas análises do artigo devido à não resposta.

## Resultados

Na primeira etapa de análise, de caráter descritivo, e com base na Tabela 1, observou-se que, do total de participantes, 255 (80,7%) utilizavam o Sistema Único de Saúde (SUS) e 226 (71,5%), serviços da rede particular. Os participantes também foram caracterizados quanto ao uso de dispositivos auxiliares de audição, sendo que 115 (36,5%) utilizavam somente o aparelho de amplificação sonora individual (AASI), 21 (6,6%) somente o implante coclear (IC) (Tabela 1) e 122 (38,6%) utilizavam ambos os tipos de dispositivo.

Na rede pública, a maioria, 164 (64,3%), considerou a comunicação ruim devido às barreiras linguísticas e comunicacionais; alguns deles ainda buscam atenuá-las oralizando, 49 (19,2%), ou escrevendo, 50 (19,6%); porém, para a maioria, 65 (25,5%), não é possível se comunicar de forma alguma.

A comunicação foi considerada boa por 91 (35,7%) participantes pelos seguintes motivos: auxílio de familiares durantes as consultas em 55 (21,6%) dos casos; presença de intérpretes de Libras em 25 (9,8%); conseguir ouvir e falar sendo usuário de AASI ou IC em 8 (3,1%) e o profissional de saúde saber Libras em 3 (1,2%) dos casos. Nas situações em que a comunicação foi avaliada como boa devido à presença de intérprete, a pesquisa quis saber se se tratava de profissionais da CIL e, portanto, profissionais habilitados conforme os requisitos legais de formação ^20^, bem como exercendo formalmente a profissão (Tabela 1).

Dentre os 25 (9,8%) que responderam contar com a ajuda de intérpretes, metade informou serem profissionais da CIL, o que leva a crer que a outra metade corresponde a intérpretes que poderiam ser profissionais ou não, informalmente desempenhando esse papel, possivelmente a pedido do usuário surdo, como uma espécie de “favor”. Cabe destacar que, dentre estes, metade (12; 50%) informou que os intérpretes acompanhantes eram da CIL, sendo que os demais alegaram não ter intérpretes disponíveis quando solicitado (8; 33,3%), não haver CIL em sua cidade (1; 4,2%), não solicitar por esquecimento (4; 4,2%) ou por não sentir necessidade (2; 8,3%).

Na rede particular, 152 (67,6%) classificaram a comunicação com os profissionais de saúde como ruim, sendo que 66 (29,3%) não conseguiam estabelecer qualquer comunicação. Para as 73 pessoas (32,4%) que avaliaram a comunicação como boa, a maior parte, 43 (19,1%), atribuiu tal facilidade ao auxílio de familiares.

Dentre os participantes que utilizavam alguma tecnologia auditiva, 77 (67%) obtiveram seus AASI e 9 (42,9%) seus IC pelo SUS, respectivamente. Nesses serviços, 43 (55,8%) dos usuários de AASI e 5 (55,6%) dos usuários de IC consideraram a comunicação ruim com os profissionais que os atenderam.

Para melhor detalhar como funciona o acesso à saúde nas redes pública e privada, segmentaram-se adicionalmente os 316 participantes entre aqueles que (1) usam ambas as redes pública e privada - 174 participantes - e (2) reportaram usar uma e somente uma das redes - 133 participantes. Nove participantes abstiveram-se ou autodeclararam não utilizar nenhuma das redes públicas, ou privadas, e suas respostas foram ignoradas.

**Figura 1:**
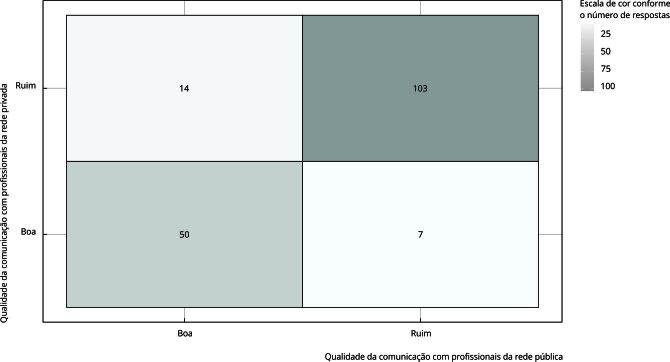
Mapa de calor da tabela de contingência da comunicação com profissionais de saúde dentre participantes usuários de ambas as redes pública e privada.

Na segunda etapa da análise, dentre os 174 participantes usuários de ambas as redes, o interesse foi atestar em que medida as avaliações da comunicação entre profissionais de saúde e participantes são concordantes. A avaliação predominante é negativa, independentemente da rede, embora a comunicação na rede privada seja pior avaliada quando comparada à da rede pública (Figura 1). 

Isso pode ser verificado tanto pelo número total de avaliações ruins em geral (117 na rede privada, contra 110 na rede pública), quanto pelo número de avaliações ruins dentre aqueles que avaliaram uma das redes com boa comunicação (14 avaliações ruins na rede privada dentre os 64 que avaliaram a rede pública com boa comunicação, contra 7 avaliações ruins na rede pública dentre os 57 que avaliaram a rede privada com boa comunicação).

Dos 174 usuários de ambas as redes, 50 (28,7%) classificam a comunicação com profissionais de saúde como boa e 103 (59,2%) como ruim, praticamente o dobro, o que indica um alto grau de concordância nas avaliações (gama de Goodman-Kruskal igual a 0,96).

Na terceira etapa de análise, para o grupo formado pelos 133 participantes autodeclarados usuários de somente uma das redes, ajustou-se um modelo de regressão logística com as variáveis explicativas rede e autoavaliação do estado de saúde, suficientes para explicar e prever a probabilidade de boa comunicação entre participantes e profissionais de saúde. Quanto às métricas de bondade de ajuste, o modelo apresentou área abaixo da curva ROC de 0,62 e atendeu ao pressuposto da normalidade dos resíduos, permitindo inferências com base nos coeficientes estimados e nas razões de chance (RC). As métricas adicionais do modelo ajustado foram deviance residual de 158,53 com 128 graus de liberdade e critério de informação de Akaike (AIC) de 164,53. Como se pode notar na Tabela 2, o modelo se ajustou bem aos dados.

**Tabela 2:** Bondade do ajuste do modelo de regressão logística com as variáveis rede e autoavaliação do estado de saúde do participante.

Rede de saúde utilizada pelo participante da pesquisa	Autoavaliação do estado de saúde	Comunicação entre participante e profissional de saúde		Predição do modelo de regressão logística para a comunicação entre participante e profissional de saúde		
		Ruim	Boa	Ruim	Boa	Probabilidade de boa comunicação entre participante e profissional de saúde (%)
Pública	Negativa	22	5	21,86	5,14	19,05
Pública	Positiva	32	22	32,14	21,86	40,47
Privada	Negativa	10	2	10,14	1,86	15,47
Privada	Positiva	25	13	24,86	13,14	34,59

Nota: o modelo logístico foi ajustado aos dados de 131 participantes com respostas completas em relação às variáveis explicativas, pois um participante não reportou se era fumante e outro participante não qualificou a comunicação com o profissional de saúde da rede privada).

O modelo final ajustado é dado pela fórmula:

$$logito(p)=log(\frac{p}{1-p})=-1,45-0,25\times {Rede}_{privada}+1,06\times {Autoavaliação}_{positiva}$$

sendo *p* a probabilidade de boa comunicação entre participante e profissional de saúde, *-1,45* o intercepto do modelo, *-0,25* o coeficiente da variável referente à utilização da rede privada de saúde e *1,06* o coeficiente da variável referente à autoavaliação positiva do estado de saúde do participante.

O uso da rede privada reduz a probabilidade de uma boa comunicação entre participante e profissional de saúde (sinal negativo), enquanto uma autoavaliação positiva de saúde do participante aumenta essa probabilidade. Mesmo no melhor dos casos, usuário da rede pública e com autoavaliação positiva de seu estado de saúde, a probabilidade de uma boa comunicação com o profissional de saúde é somente de 40,47%, ratificando os problemas de comunicação enfrentados pelos surdos usuários de Libras no acesso à saúde (Tabela 2).

Exponenciando-se os coeficientes do modelo de regressão logística, obtêm-se as RC ^18^, que permitem concluir que, controlando para a autoavaliação do estado de saúde, surdos usuários de Libras e unicamente da rede privada de saúde têm 22% menos chances de ter uma boa comunicação com o profissional de saúde (RC ~ 0,79) quando comparados com aqueles que frequentam unicamente a rede pública. Da mesma forma, controlando para a rede de saúde utilizada, surdos usuários de Libras e com autoavaliação positiva de saúde têm quase três vezes mais chances de ter uma boa comunicação com o profissional de saúde (RC ~ 2,89).

Na quarta etapa de análise, e devido à estrutura relevante dos serviços de saúde para a Região Metropolitana de Campinas, à disponibilidade da CIL e à maior concentração de participantes da pesquisa, Campinas foi escolhida como foco da análise descritivo-espacial.

**Figura 2:**
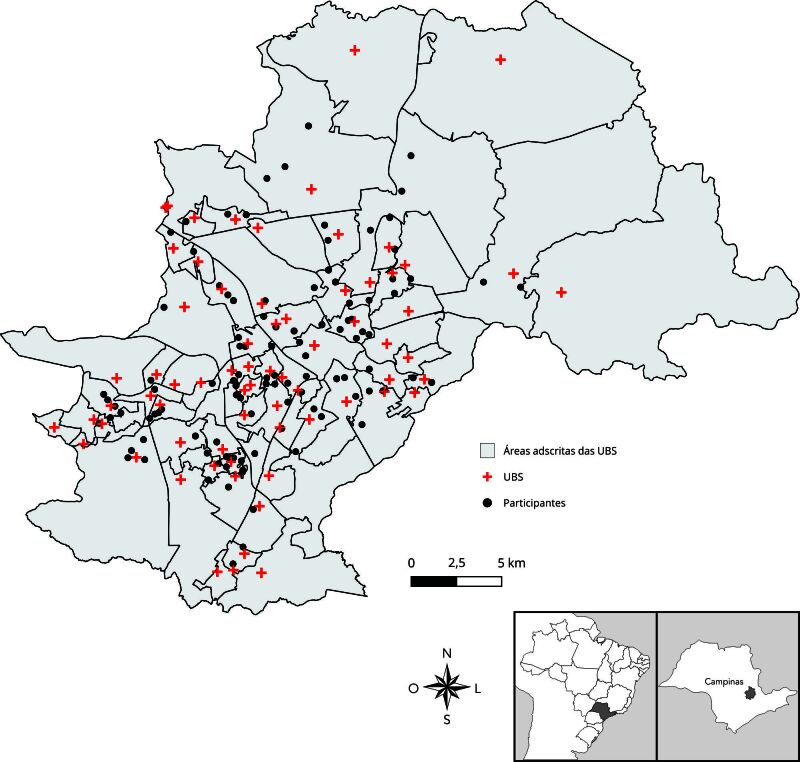
Distribuição dos participantes da pesquisa em Campinas, São Paulo, Brasil, nas respectivas áreas adscritas das unidades básicas de saúde (UBS).

Dos 316 participantes da Região Metropolitana de Campinas que autodeclararam seu CEP de forma válida, 142 residiam em Campinas. Ao todo, 145 participantes informaram residir em Campinas, mas três não informaram seu CEP e, portanto, não fizeram parte da análise. A Figura 2 ilustra a distribuição dos participantes da pesquisa em Campinas nas respectivas áreas adscritas das UBS.

Nota: dos 145 participantes de Campinas, somente 142 informaram seu CEP de maneira válida e estão contemplados neste mapa.

As UBS com o maior número de participantes pertencentes às suas áreas adscritas foram Dic III, com 9 respostas, seguida por Guanabara e Aurélia, com 8 cada, e Centro e Florence, com 6 cada, tendo todas as demais menos de 5 respostas (Quadro 1). Agrupando o número de respostas por Distritos de Saúde, tem-se que o Distrito de Saúde Sudoeste responde pelo maior número de participantes (35), seguido por Leste (27) e Norte (27), Noroeste (22), Sul (18) e Sudeste (13), respectivamente (dados não apresentados em tabela).

**Quadro 1:** Densidade de participantes por áreas adscritas das unidades básicas de saúde (UBS).

UBS (DISTRITO DE SAÚDE)	PARTICIPANTES
Dic III (SO)	9
Aurélia (N), Guanabara (L)	8
Centro (L), Florence (NO)	6
Campos Elíseos (SO), Dic VI (SO), Figueira (S)	5
Barão Geraldo (N), Boa Vista (N), Esmeraldina (SL), Integração (NO), União dos Bairros (SO), Valença (NO), Vila União (SO)	4
Carvalho de Moura (S), Cássio Raposo (N), Conceição (L), Costa e Silva (L), Nova América (S), São Marcos (N), São Quirino (L), Taquaral (L), Vila Ipê (SL)	3
Aeroporto (SO), Anchieta (N), Campo Belo (S), Eulina (N), Ipaussurama (NO), Pedro de Aquino (NO), Santa Odila (SL), São Bernardo (SL), Sousas (SL), Vila Rica (S), Vista Alegre (SO)	2
31 de Março (L), Capivari (SO), Dic I (SO), Itajaí (NO), Lisa (NO), Oziel (S), Rossin (NO), Santa Bárbara (N), Santa Lúcia (SO), Santo Antônio (SO), Santos Dumont (S), São José (S), Satélite Íris I (NO)	1
Bassoli (NO), Campina Grande (NO), Carlos Gomes (L), Fernanda (S), Floresta (NO), Joaquim Egídio (SL), Orosimbo Maia (SL), Paranapanema (SL), Perseu (NO), Rosália (N), San Diego (S), San Martin (N), Santa Mônica (N), Santa Rosa (NO), São Cristóvão (SO), São Domingos (S), São Vicente (SL), Vicente Pisani (NO), Village (N)	0

Nota: dos 145 participantes de Campinas, somente 142 informaram seu CEP de maneira válida e estão contemplados nesta análise.

Distritos de Saúde: L: Leste, N: Norte, NO: Noroeste, S: Sul, SO: Sudoeste, SL: Sudeste.

## Discussão

Sob técnicas e pontos de vista distintos, a análise do acesso à saúde e da qualidade de comunicação com os profissionais em serviços públicos e privados apontou que, tanto nos atendimentos no SUS quanto nos serviços particulares de saúde, há barreiras linguísticas e comunicacionais, sobretudo pelo desconhecimento da Libras pelos profissionais.

O SUS obteve uma melhor avaliação comparado aos serviços privados, uma vez que, controlando para a autoavaliação do estado de saúde, surdos usuários de Libras e unicamente da rede pública apresentaram 22% mais chances de ter uma boa comunicação com o profissional de saúde quando comparados com aqueles que frequentam unicamente a rede privada. Uma minoria dos usuários, tanto na rede privada (0,4%) quanto na rede pública (1,2%), respondeu que é atendida por profissionais de saúde que sabem Libras, o que afetou diretamente a forma como esses indivíduos avaliaram a comunicação.

Alguns estudos ^11^ ^,^ ^21^ apontam para a escassez de profissionais de saúde aptos a trabalhar com a população surda, o que foi corroborado na presente pesquisa, na medida em que a probabilidade de haver uma boa comunicação com o profissional é de somente 40,47%, no melhor dos casos (usuário da rede pública e com autoavaliação positiva de seu estado de saúde). Segundo pesquisa que investigou a postura de trabalhadores da área da saúde chilenos em relação à surdez, dos profissionais incluídos no estudo, apenas uma pequena parcela tinha treinamento adequado para atender pacientes surdos e poucos profissionais haviam feito curso em língua de sinais em algum momento da formação, embora, aproximadamente, 80% deles já tivessem atendido usuários Surdos em algum momento de sua atuação ^11^.

Esse problema não só atinge a dinâmica entre profissional-paciente, mas também engloba a relação entre indivíduo surdo e sistema de saúde, como demonstra um relato de experiência de trabalhadores da saúde em uma unidade de saúde da família (USF) na Bahia que evidencia a falta de estabelecimento de vínculo entre a USF e o paciente surdo ^7^.

Uma vez que a maioria das unidades de atendimento é desprovida de ferramentas de inclusão, os surdos usuários de Libras frequentemente se veem obrigados a buscar outras formas de comunicação, como a linguagem escrita, oralização, leitura orofacial, auxílio de acompanhante ou intérprete, realização de gestos, entre outros ^13^.

Quando são colocadas em evidência as respostas dos indivíduos que classificaram a comunicação como boa, percebe-se que a maior parte avaliou dessa maneira não pelo fato de o profissional de saúde saber Libras, mas sim porque obteve a ajuda de terceiros, como familiar ou intérprete de língua de sinais. De acordo com diversos estudos ^3^ ^,^ ^13^ ^,^ ^21^ ^,^ ^22^, os surdos consideram tais estratégias, que não envolvem o diálogo direto do paciente com o profissional, ineficientes para uma comunicação efetiva, porque dificultam uma escuta qualificada das suas necessidades de saúde.

Se, por um lado, a mediação de intérprete ou familiar pode ser decisiva para a prestação do atendimento, por outro, evidencia a falta de autonomia e privacidade do surdo no cuidado de sua própria saúde ^2^, afinal, a comunicação é estabelecida entre os ouvintes sem que haja a participação plena do paciente ^3^.

Essas ocorrências os afastam dos serviços de saúde, pois, para não vivenciarem situações como essas, marcadas por constrangimento, desconforto e frustração ^13^ ^,^ ^21^, eles acabam diminuindo suas idas aos atendimentos ^3^ ^,^ ^22^ ^,^ ^23^.

Ainda assim, a presença de intérpretes nos atendimentos configura uma alternativa melhor se comparada à impossibilidade de comunicação. No Brasil, existe um serviço gratuito chamado CIL, fomentado por uma política de equipagem lançada em 2013 pela Secretaria de Direitos Humanos da Presidência da República.

A CIL visa à promoção da acessibilidade linguística nos órgãos públicos por meio da ação de TILS. Essas instituições estão presentes em diversas cidades do país, sendo que, na Região Metropolitana de Campinas, o único município que conta com uma unidade é Campinas, que, por vezes, atende pessoas que não moram especificamente nesta cidade.

É notável que, mesmo entre a pequena parcela de surdos que têm acesso a intérpretes de Libras em suas consultas, apenas metade deles é atendida por profissionais da CIL, enquanto os outros contam com intérpretes de seu círculo pessoal. A profissão de TILS, embora tenha sido regulamentada em 2010, ainda é estigmatizada por ser associada à caridade e ao assistencialismo, muitas vezes sendo exercida por indivíduos sem formação específica e de forma voluntária ^24^. Tal situação não apenas contribui para a desvalorização da profissão, mas também pode comprometer a qualidade dos serviços de saúde, aumentando o risco de erros de diagnóstico, falta de garantia de confidencialidade, entre outros problemas.

Vale ressaltar que, quando da coleta de dados desta pesquisa, a CIL contava com três profissionais, mas, em 2023, o quadro se ampliou para nove TILS, o que certamente já deve ter resultado em melhora da acessibilidade.

A limitação das competências dos profissionais de saúde para lidar com as especificidades do atendimento à comunidade surda não é uma realidade exclusiva do Brasil, mas sim um problema global, como mostram estudos da Inglaterra ^10^, Grécia ^25^, África Sub-saariana ^26^, Nigéria, Estados Unidos, Países Baixos e Israel ^22^.

Como exemplo, foi reportado que pacientes surdos nos Estados Unidos são menos propensos a confiarem no serviço e entenderem o diagnóstico e tratamento em relação aos ouvintes ^22^. Na Grécia, estudo afirma que pessoas com deficiência auditiva são sistematicamente excluídas das políticas públicas de saúde ^25^, mesmo que haja uma tendência global de busca por melhoria na sua qualidade de vida.

A implementação de cursos obrigatórios que abordem as temáticas de cultura surda e língua de sinais nos cursos da área da saúde é sugerida, pois seria um facilitador do cuidado de pessoas surdas ^27^. Contudo, no Brasil ainda há de se percorrer um longo caminho para que esse preceito seja realidade. Um estudo publicado em 2020 evidenciou que, apesar de 43,1% dos cursos de graduação na área da saúde em instituições de Ensino Superior brasileiras oferecerem disciplina de Libras, a maioria (83,3%) a oferece como disciplina optativa ^28^ e apenas 16,7% como obrigatória.

Mesmo nas instituições em que o ensino da Libras é oferecido, destacaram-se diversos problemas relacionados à forma como era colocado em prática, seja por carga horária ineficiente destinada ao ensino da disciplina, seja por educação focada no vocabulário e gramática em detrimento do enfoque na inclusão e compreensão dos aspectos culturais da população surda ^29^. Conclui-se que não basta que os cursos apenas incluam a Libras em sua grade curricular; deve-se prezar também pela excelência do ensino para que a mudança possa reverberar positivamente no atendimento em saúde ao indivíduo surdo.

No presente estudo, foi constatado que a maioria dos surdos não faz uso de dispositivos auxiliares de audição. O AASI foi a tecnologia mais utilizada dentre os participantes quando comparado ao IC. Esses dados estão em conformidade com um trabalho que investigou a prevalência de doenças crônicas não transmissíveis em pessoas surdas da Região Metropolitana de Maringá, no Paraná, o qual reportou que mais da metade dos participantes não fazia uso de próteses, 30,9% utilizavam AASI e 1,8% tinham IC ^23^.

Além disso, nesta pesquisa, foi observado que a maior parte dos surdos que possuem AASI o obteve pelo SUS (61,4%), ao passo que o IC foi obtido predominantemente na rede privada, uma vez que 60,9% dos participantes relataram não terem obtido o IC por meio do serviço público. Seria interessante que a ampliação da oferta de IC fosse alvo de investimento pela administração pública, visto que um estudo que investigou a satisfação e qualidade de vida em usuários de IC com longo tempo de privação sensorial concluiu que os participantes apresentaram alto nível de satisfação com seu uso ^30^.

Entre as pessoas que obtiveram AASI pelo SUS, a porcentagem de atendimento por profissionais que sabem Libras (8,6%) foi maior em relação à porcentagem reportada na comunicação com profissionais da rede pública (1,1%) e privada (0,4%). Desse dado, é possível inferir que, nos atendimentos em saúde da rede pública voltados especificamente à população com deficiência auditiva, há mais profissionais com conhecimento na área e, portanto, maior acessibilidade linguística e comunicacional.

Em relação à análise geoespacial, o mapa (Figura 2) mostra a distribuição de UBS por todo o território de Campinas, mas isso por si só não garante o acesso da população surda a elas, devido às dificuldades de acessibilidade comunicacional e linguística e ao fato de a CIL não conseguir atendê-la. O mapeamento produzido neste estudo pode servir de subsídio para que as unidades de saúde tomem conhecimento da demanda dessa população e, ao considerar a espacialidade da população surda, façam um melhor uso da estrutura pública de atendimento aos direitos desta população e suas especificidades linguísticas.

Em abril de 2024, a gestão municipal de Campinas lançou o Programa Acessa Libras, que disponibiliza uma Central de Libras remota para atendimento de surdos em serviços públicos municipais, incluindo UBS, 24 horas por dia, nos sete dias da semana. Por meio desse recurso, um usuário surdo que vai até a UBS pode acionar a Central de Libras via código QR, por meio do seu próprio celular, para mediar a comunicação entre ele e o profissional de saúde ^31^. O desafio que se impõe é que, por ser um programa recente, poucos usuários e profissionais conhecem esse recurso.

Como limitações inerentes ao estudo, pode-se citar a forma de coleta de dados, cujo caráter on-line geralmente está associado a maiores taxas de não resposta e pressupõe um nível socioeconômico do participante que viabilize seu acesso à internet de banda larga e equipamentos eletrônicos. Ademais, o caráter intencional e não probabilístico da amostragem impede a generalização das conclusões para além da amostra e local do estudo. Sem deixar de considerar restrições logísticas e orçamentárias, como abordagem complementar para alcançar surdos sem acesso à internet em futuros estudos, recomenda-se o emprego de entrevistas presenciais realizadas por entrevistadores fluentes em Libras ou com mediação de TILS.

## Conclusões

O estudo evidenciou graves limitações de acesso à saúde de surdos usuários de Libras, com 18 anos ou mais, moradores da Região Metropolitana de Campinas, em função de barreiras linguísticas e comunicacionais com profissionais de saúde das redes de saúde pública e privada, esta última pior avaliada.

São necessários mais investimentos voltados à superação das barreiras identificadas e promoção de acessibilidade. Recomenda-se a implementação de um conjunto de medidas amplo, como: contratação de mais TILS para a CIL existente, preferencialmente por meio de concursos públicos, com salários justos; criação de CIL nas demais cidades da Região Metropolitana de Campinas; oferta de cursos de Libras para profissionais da rede SUS como parte da educação permanente; promoção do ensino de Libras em todos os cursos da área da saúde nas universidades.

Para tanto, é fundamental uma forte mobilização social e decisão política que considere que o SUS deve ser universal, integral e equânime também para a população surda.
